# Neurobiological mechanisms of acupuncture for post-ischemic stroke comorbid insomnia and cognitive impairment: a narrative review

**DOI:** 10.3389/fneur.2026.1696958

**Published:** 2026-02-25

**Authors:** HuiHui Yin, Ming Liu, Ce Shi, JiaXi Liu, Xia Sun, XianFeng Ye

**Affiliations:** 1 Third Clinical Medical College, Henan University of Chinese Medicine, Zhengzhou, Henan, China; 2 Department of Rehabilitation, The Third Affiliated Hospital of Henan University of Chinese Medicine, Zhengzhou, Henan, China

**Keywords:** acupuncture, BDNF–TrkB-PI3K/Akt pathway, brain network remodeling, cognitive impairment, insomnia, neurobiological mechanisms, post-ischemic stroke, TCM syndrome differentiation

## Abstract

This narrative review systematically synthesizes recent clinical and pre-clinical evidence to elucidate the latest neurobiological mechanisms underlying acupuncture for post-stroke insomnia combined with cognitive impairment (PS-ICI). PS-ICI is characterized pathologically by a hippocampal–prefrontal circuitry-mediate “sleep–cognition vicious cycle” and clinically by concurrent cognitive decline and sleep-architecture disruption, both of which markedly impede post-stroke neurological recovery. Grounded in the Traditional Chinese Medicine (TCM) principle of “regulating Shen and re-animating the brain, “acupuncture exerts bidirectional modulation on cognition and sleep, significantly improving core functional outcomes and activities of daily living. Up-to-date studies confirm that synergistic, multi-dimensional effects are achieved through regulation of the BDNF–TrkB–PI3K/Akt signaling axis, preservation of neurovascular unit integrity, restoration of gut–brain axis homeostasis, normalization of circadian immune rhythms, and reshaping of default-mode network (DMN) plasticity. Given the high heterogeneity of included studies, a qualitative integrative approach was employed. Current evidence is nevertheless limited by small sample sizes, short follow-up durations, and substantial heterogeneity in acupuncture parameters (frequency and point selection); future work must therefore focus on dissecting inter-pathway interactions, standardizing therapeutic protocols, and integrating multi-omic technologies to propel acupuncture toward precision, evidence-based management of PS-ICI.

## 1 Introduction

Ischemic stroke (IS) is a leading global cause of long-term disability and mortality (1). Although hyper-acute management has become increasingly effective, long-term care after stroke is still challenged by “neuro-psychiatric comorbidities.” Epidemiological surveys show (2) that 30–60% of IS survivors develop post-stroke insomnia within 3–12 months after onset; more than half of these individuals concurrently experience varying degrees of cognitive decline, constituting the clinical phenotype of PS-ICI. The core pathological feature of PS-ICI is a “hippocampal-prefrontal circuitry-mediated vicious cycle of sleep-cognition interaction.” Research indicates (3) that by post-stroke day 14 patients’ sleep efficiency is <65%, and at 1 year the Montreal Cognitive Assessment (MoCA)—used to screen for mild cognitive impairment (MCI) and to evaluate global cognition—declines by an average of 3.8 points, with particularly pronounced damage in the domains of delayed recall, abstract reasoning, and executive function. Each additional week of insomnia increases the risk of cognitive deterioration by 12%. This comorbidity not only markedly delays neurological recovery, but also raises the risk of stroke recurrence, depression, and anxiety, while lowering quality of life and doubling health-care utilization. Modern medicine has attempted pharmacological and non-pharmacological interventions such as benzodiazepines, melatonin-receptor agonists, cognitive-behavioral therapy for insomnia, and donepezil (4); however, these are limited by poor drug tolerability, drug–drug interactions, and polypharmacy contraindications after stroke, and they offer only modest capacity to restore the reciprocal “cognition-sleep” network, underscoring the urgent need to integrate complementary and alternative medical strategies.

As a core external therapy in TCM, acupuncture is centered on the theory of “regulating the mind and awakening consciousness,” first documented in the classic TCM text Lingshu·Da Huo Lun: “Defensive qi fails to enter the yin (interior) and remains in the yang (exterior). When it lingers in the yang, yang qi becomes excessive, and the yang heel vessel (yangqiao mai) flourishes; when it cannot enter the yin, yin qi becomes deficient—thus the eyes cannot close (insomnia); mental disturbance leads to forgetfulness” (5). This lays a solid theoretical foundation for acupuncture in treating PS-ICI. Acupuncture offers advantages such as holistic regulation, broad target coverage, and minimal side effects. In recent years, evidence from neuroimaging, electrophysiology, and molecular biology has demonstrated that acupuncture modulates multiple dimensions including the “gut-brain axis” (6), “neurovascular unit” (7), and “glymphatic system” (8). It improves microcirculation in the ischemic penumbra, remodels neural network plasticity, thereby enhancing sleep architecture and promoting cognitive function recovery. Additionally, acupuncture downregulates proinflammatory cytokines [e.g., interleukin-1β (IL-1β), tumor necrosis factor-*α* (TNF-α)] and upregulates neurotrophic factors [Brain-Derived Neurotrophic Factor (BDNF), Insulin-like Growth Factor-1(IGF-1)] (9), alleviating post-stroke neuroinflammation and oxidative stress—providing a biological basis for the concurrent intervention of PS-ICI. However, most existing studies focus on single outcome measures, lacking systematic elaboration of the dual-axis interactive mechanisms between sleep and cognitive impairment in PS-ICI (10). Moreover, challenges such as small sample sizes, high heterogeneity in intervention protocols, and short follow-up durations hinder the integration of clinical evidence into evidence-based consensus. Against this backdrop, this review adopts a holistic perspective of the “post-stroke neural plasticity-sleep-cognition network,” synthesizing clinical and basic research advances in acupuncture for PS-ICI over the past 6 years. Its aim is to systematically organize relevant evidence and dissect the core neurobiological mechanisms. Given the high heterogeneity of included studies, this narrative review employs a qualitative synthesis approach.

## 2 Research methods

### 2.1 Search strategy

This is a narrative review. We systematically searched for relevant studies published from January 2019 to December 2025 across the following databases: PubMed, Web of Science, Embase, China National Knowledge Infrastructure (CNKI), Wanfang Data, and VIP Chinese Science and Technology Journal Database. The English search term combination was: (“Ischemic Stroke” OR “Cerebral Infarction”) AND (“Insomnia” OR “Sleep Disturbance”) AND (“Cognitive Impairment” OR “Dementia”) AND (“Acupuncture” OR “Electroacupuncture” OR “Scalp Acupuncture”). Corresponding Chinese search terms were used for the Chinese databases. Additionally, we manually searched the references of included studies and relevant reviews to avoid missing eligible literature.

### 2.2 Inclusion and exclusion criteria

Inclusion Criteria: ① Definite diagnosis of PS-ICI with clear diagnostic criteria: ischemic stroke per WHO criteria, insomnia per DSM-5 criteria, and TCM syndrome differentiation explicitly defined; ② acupuncture as the core intervention with detailed documentation of acupoints, operational parameters (e.g., electroacupuncture frequency 2–100 Hz), and treatment course (≥2 weeks); ③ clinical studies [randomized controlled trial (RCT), cohort study] or basic experimental studies; ④ core outcome measures: clinical studies must include cognitive assessment via MoCA/Loewenstein Occupational Therapy Cognitive Assessment (LOTCA)/Mini-Mental State Examination (MMSE) and sleep-quality assessment via Pittsburgh Sleep Quality Index (PSQI)/Athens Insomnia Scale (AIS)/polysomnography (PSG); ⑤ studies published in English or Chinese.

**Figure 1:**
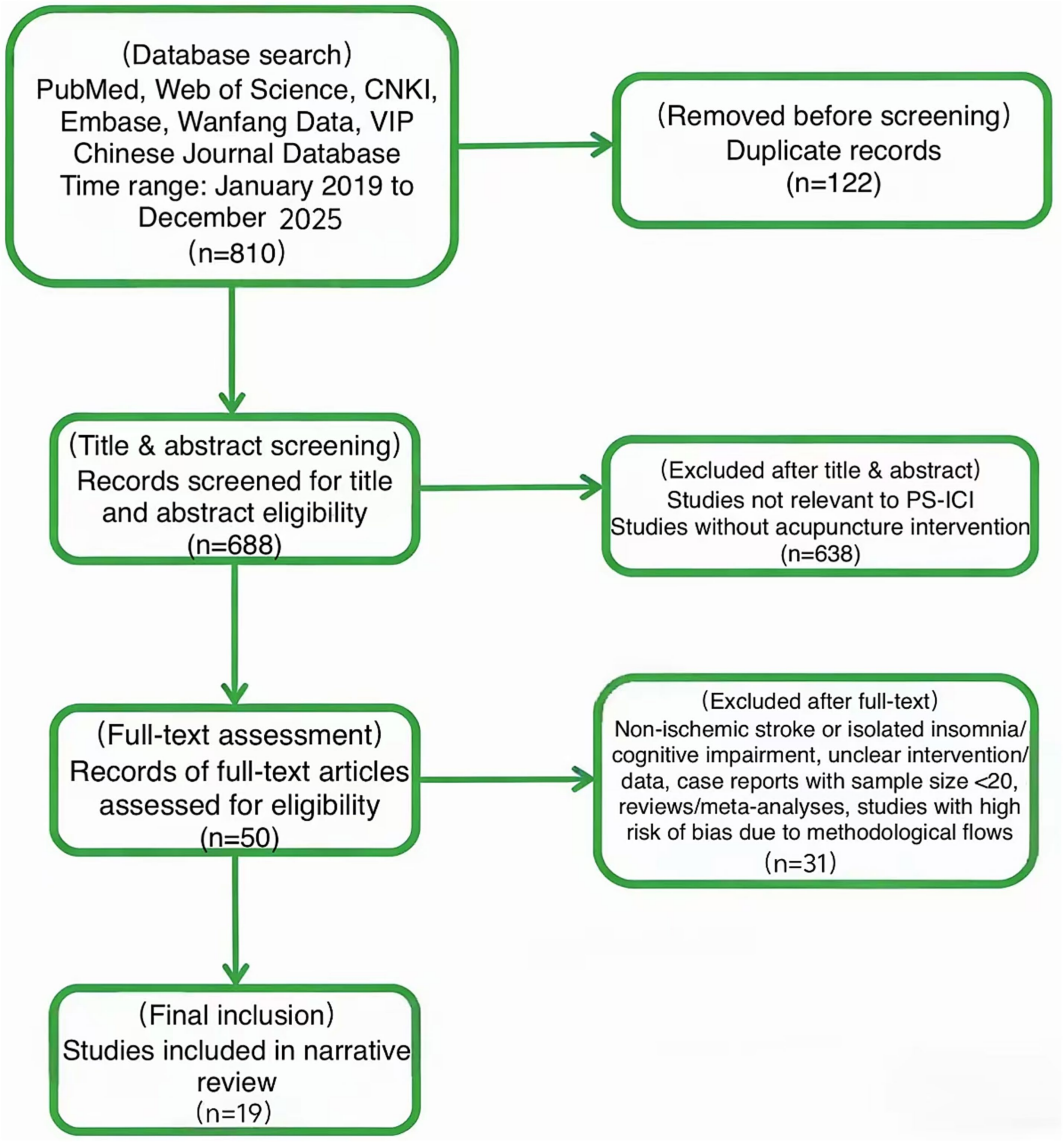
PRISMA flow diagram of literature search and screening process. This figure delineates the stepwise screening trajectory for studies evaluating acupuncture in PS-ICI. Key phases—database retrieval, duplicate removal, title/abstract screening, and full-text adjudication—are displayed together with the exact number of records retained at each node. Exclusion criteria (e.g., irrelevant interventions, non-ischemic stroke cohorts) and the final count of articles meeting eligibility are explicitly annotated.

Exclusion Criteria: ① Non-ischemic stroke or isolated insomnia/cognitive impairment (no comorbidity); ② intervention protocol unclear or data incomplete; ③ case reports with sample size < 20; ④ high risk of bias due to serious methodological flaws (e.g., RCT without concealed randomization). A total of 810 potentially relevant records were identified in the initial search; 791 records were excluded after applying the above criteria, leaving 19 studies for final inclusion. The literature search and screening flowchart is presented in Figure 1.

### 2.3 Characteristics of included studies

After a systematic search that initially yielded 810 records, we ultimately included 19 studies—18 independent clinical trials and 1 narrative review on auricular acupuncture—comprising 2,842 patients with PS-ICI. The review article was used to supplement evidence on the specific modality. All investigations were conducted in China. Among the 18 clinical trials, illness-stage distribution was acute (*n* = 1), subacute (*n* = 6), subacute-to-chronic (*n* = 7), chronic (*n* = 1), and unspecified (*n* = 3). Participant age ranged from 30 to 80 years, with the majority being middle-aged and older adults (45–78 years). In the 7 trials that reported complete sex data, the male-to-female ratio was approximately 1.2:1. TCM syndrome differentiation was explicitly documented in 6 trials (liver–kidney yin deficiency, liver-qi stagnation, binding depression of liver-qi, heart–spleen deficiency, heart–kidney non-interaction, and phlegm-heat intestinal excess), whereas 12 trials did not specify or report TCM patterns. However, the associations between TCM patterns and MoCA/PSQI scores or BDNF levels remain undefined, and large-scale data linking patterns to neurobiological phenotypes are lacking.

### 2.4 Bias-risk assessment

Two independent reviewers (Yin Huihui and Liu Jiaxi) applied the Cochrane Risk-of-Bias 2.0 tool to evaluate all 18 randomized controlled trials. The assessment covered six domains, including random-sequence generation and allocation concealment; each trial was rated as having a low, moderate, or high risk of bias. Disagreements were resolved by consultation with a third reviewer (Ye Xianfeng) to ensure objectivity and accuracy. Only studies published within the past 6 years (January 2019 – December 2025) were included (Table 1).

**Table 1:** Risk of bias assessment for included randomized controlled trials.

Study (First Author, Year, Reference No.)	Random Sequence Generation	Allocation Concealment	Blinding of Participants and Personnel (Performance Bias)	Blinding of Outcome Assessment (Detection Bias)	Incomplete Outcome Data (Attrition Bias)	Selective Reporting	Overall Risk of Bias
Liu J, 2025, [13]	Low	Unclear	High	Low	Low	Low	Moderate
Yang Y, 2025, [14]	Low	Unclear	High	Low	Low	Low	Moderate
Zhang C, 2021, [16]	Low	Low	High	Low	Low	Low	Moderate
Zhang S, 2022, [17]	Low	Low	High	Low	Low	Low	Moderate
Jiao M, 2024, [19]	Low	Low	Low	Low	Low	Low	Low
Wang D, 2019, [20]	Low	Unclear	High	Low	Low	Low	Moderate
Sui S, 2020, [23]	Low	Unclear	High	Low	Low	Low	Moderate
Yin Z, 2022, [29]	Low	Unclear	High	Low	Low	Low	Moderate
Liu W, 2025, [30]	Low	Unclear	High	Low	Low	Low	Moderate
Zhang P, 2021, [35]	Low	Unclear	High	Low	Low	Low	Moderate
Shi S, 2020, [36]	Low	Unclear	High	Low	Low	Low	Moderate
Bian Z, 2025, [43]	Low	Unclear	High	Low	Low	Low	Moderate
Zhang T, 2025, [44]	Low	Unclear	High	Low	Low	Low	Moderate
Li N, 2023, [50]	Unclear	Unclear	High	Unclear	Low	Unclear	High
Zhou N, 2025, [51]	Low	Unclear	High	Low	Low	Low	Moderate
Cai X, 2022, [59]	Unclear	Unclear	High	Unclear	Low	Unclear	High
Zhang BY, 2024, [61]	Low	Low	Low	Low	Low	Low	Low
Wang R, 2023, [63]	Low	Low	Low	Low	Low	Low	Low

This table was constructed using the Cochrane Risk of Bias Assessment Tool (RoB 2.0). The rating definitions are as follows: Low risk = low risk of bias; Unclear = insufficient methodological information to determine the risk of bias; High risk = high risk of bias. The overall risk of bias was comprehensively determined based on the most conservative rating across all assessment domains, which is consistent with the methodological standards for narrative reviews.

## 3 Clinical research on acupuncture treatment for PS-ICI

Studies have shown that acupuncture improves patients’ cognitive function and sleep quality through multiple mechanisms, including the regulation of neural signaling pathways, neurovascular protection, immune modulation, and brain network regulation (Table 2).

**Table 2:** Summary table of acupuncture interventions for PS-ICI.

Chapter number	Acupuncture type	Study type	Sample size	Intervention stage	Intervention type	Control measure	TCM syndrome type	Primary outcomes (scale/indicator + result)	Country	Main limitations	Mortality/dependence status
3.1	(13) Body Acupuncture	Single-center RCT	108 cases	Subacute to chronic (2–12 months)	Observation: Tongdu Jieyu Acupuncture + Tongqiao Jieyu Decoction + conventional treatment Control: Western medicine (Sertraline) alone	Conventional Western medicine (no sham acupuncture)	Liver-Kidney Yin Deficiency	1. Cognition: RBANS (observation group better in all dimensions) 2. Sleep: PSQI (9.06 ± 1.08, better than control) 3. Depression: HAMD (5.24 ± 0.65, better than control)	China	No sham acupuncture control	Not reported
3.1	(14) Body Acupuncture	Single-center RCT	60 cases	Subacute to chronic (2–6 months)	Experimental: Acupuncture at Back-Shu Points + “Back-She Points” + individualized treatment Control: Conventional acupuncture + individualized treatment	Conventional acupuncture (no sham acupuncture)	Not specified	1. Cognition: MoCA (experimental group better) 2. Daily ability: ADL (81.78 ± 1.33, better than control)	China	1. Small sample 2. No sham control 3. Lack long-term follow-up	Not reported
3.2	(16) Scalp Acupuncture	Multi-center RCT	445 cases	Subacute to chronic (30–180 days)	Experimental: Conventional medicine + rehab + interactive scalp acupuncture (synchronous cognitive training) Control: Conventional medicine + rehab + conventional scalp acupuncture (phased training)	No sham acupuncture	Stroke-Middle Meridian (not subdivided)	1. Cognition: MoCA/MMSE increased 2. Sleep: PSQI decreased 3. Neurotrophic factors: BDNF/NGF increased	China	1. No sham control 2. Some patients lost to follow-up	Not reported; Experimental: Basic self-care (MBI 60–100)
3.2	(17) Scalp Acupuncture	Multi-center RCT	660 cases	Subacute to chronic (15–180 days)	3 groups: Interactive scalp acupuncture/Simple scalp acupuncture/Scalp acupuncture + cognitive training (all + medicine + exercise)	Simple scalp acupuncture; Phased training	Stroke-Middle Meridian (not subdivided)	Cognition: MoCA (interactive group best)	China	1. Institutions limited to Shenzhen 2. Incomplete double-blinding	Not reported
3.3	(19) Electroacupuncture	RCT	36 cases	Chronic (3 months–1 year)	Electro group: Electroacupuncture at Sishencong + cerebral infarction treatment Sham group: Sham acupuncture at non-acupoints + same treatment	Sham acupuncture	Not reported	1. Sleep: PSQI/PSG improved 2. Cognition: MoCA-B/STM coding test improved	China	1. Small sample 2. No brain mechanism exploration	Not reported
3.3	(20) Electroacupuncture	RCT	60 cases	Subacute (2 weeks–6 months)	Electro group: Electroacupuncture at Sishencong + cerebral infarction treatment Control: Oral Estazolam + same treatment	Oral Estazolam (no sham)	Not reported	1. Sleep efficiency: Improved (CPC evaluated) 2. Cognition: MoCA (nomenclature/attention improved)	China	1. No sham control 2. Limited sample	Not reported
3.4	(22) Ear Acupuncture	Review	60 cases	Not specified	Not reported (summarized past methods)	Not reported	Yin Deficiency; Phlegm-Stasis Interbinding (summarized)	Not reported (summarized past conclusions)	China	Not reported	Not reported
3.5	(24) Acupuncture + Medicine	RCT	80 cases	Subacute (~30 days)	Combined: Tiaoren Tongdu Acupuncture + Huoxue Jieyu Decoction + Western medicine Control: Western medicine alone	Western medicine (no sham)	Not subdivided (PSCI: Spleen-Kidney Yang Deficiency; Insomnia: Liver-Qi Stagnation)	1. Cognition: LOTCA improved 2. Sleep: PSQI improved	China	1. No sham control 2. Limited sample	Not reported
4.1	(30) Acupuncture + rTMS	RCT	90 cases	Subacute to chronic (3–12 months)	3 groups: Acupuncture + rTMS + medicine/rTMS + medicine/medicine alone (Escitalopram)	No sham acupuncture	Liver-Qi Stagnation	1. Cognition: MoCA (27.87 ± 2.15, best) 2. Sleep: PSQI (7.43 ± 2.15, best)	China	1. Small sample 2. Short observation 3. No sham stimulation	Not reported
4.1	(23) Acupuncture + TCM	RCT	80 cases	Recovery (37–58 days)	Acupuncture group: Tiaohe Acupuncture + Estazolam Combined group: Acupuncture + Shugan Jieyu Decoction + Estazolam	Estazolam + Tiaohe Acupuncture (no sham)	Liver-Qi Stagnation	Sleep: PSQI (combined group better)	China	1. No sham control 2. No cognition/dependence evaluation	Not reported
4.2	(35) Acupuncture + Western Medicine	Prospective RCT	88 cases	Subacute/chronic not specified	Observation: Medicine + acupuncture Control: Medicine alone (Edaravone/Xuesaitong)	Medicine alone (no sham)	Not reported (lethargy/snoring)	1. Sleep: PSG improved 2. Cognition: MoCA improved	China	1. Single-center 2. Stage not specified	Not reported
4.2	(36) Scalp Acupuncture + Transcranial Ultrasound	Not specified	122 cases	Acute (within 2 weeks)	Experimental: Ultrasound + scalp acupuncture Control: Ultrasound alone	Ultrasound alone (no sham)	Not reported	1. Sleep: PSQI improved 2. Neurological function: NIHSS decreased	China	1. No blinding 2. Short follow-up (30 days)	Not reported
4.3	(43) Tiaoshen Xingnao Acupuncture	RCT	128 cases	Subacute to chronic (6.17–6.34 months)	Observation: Tiaoshen Xingnao Acupuncture + Estazolam + conventional treatment Control: Estazolam + conventional treatment	No sham acupuncture	Heart-Spleen Deficiency	1. Cognition: MoCA-B (23.14 ± 2.49, better than control) 2. Sleep: PSQI/AIS decreased	China	1. No sham control 2. No blinding 3. Short observation	Not reported
4.3	(44) Acupuncture + Western Medicine	Prospective RCT	85 cases	Subacute to chronic not specified	Observation: Acupuncture + Butylphthalide Control: Butylphthalide alone	Butylphthalide alone (no sham)	Heart-Kidney Disharmony	1. Sleep: PSQI/ISI improved 2. Cognition: MoCA improved	China	1. No sham control 2. Lack long-term follow-up	Not reported
4.4	(50) Manual Acupuncture	RCT	216 cases	Not specified	Manual acupuncture (Zusanli/Baihui/Xuehai): Twice a week, 30 min/session, 12 weeks	Only vs. healthy controls (no sham)	Not reported	1. Cognition: MoCA (32%↓ before; 29%↑ after) 2. Sleep: PSQI (38%↑ before; 31%↓ after)	China	1. No sham control 2. Healthy control number unknown 3. No long-term efficacy	Not reported
4.4	(51) Acupuncture + TCM + Western Medicine	RCT	220 cases	Recovery (1–4 months)	Experimental: Huanglian Wendan Decoction + acupuncture + medicine + Estazolam Control: Medicine + Estazolam	Medicine + Estazolam (no sham)	Phlegm-Heat Obstructing Fu-Organ	1. Sleep: AIS improved 2. Cognition: MoCA improved	China	1. No sham control 2. Single-center limitation	Not reported
4.5	(59) Acupuncture + HBO + Rehab	RCT	196 cases	Not reported	Observation: Conventional treatment + rehab + HBO + Zhishen Three Acupoints Control: Conventional treatment + rehab + HBO	No sham acupuncture	Not reported	1. Cognition: MoCA/LOTCA improved 2. Sleep: PSQI/AIS decreased	China	Not reported	Not reported
5.1	(61) Electroacupuncture	Mixed study (A: RCT; B: Non-randomized)	72 cases	Subacute (3–12 months)	Electro group: Low-frequency (2 Hz) electroacupuncture Sham group: Sham acupuncture at non-meridian points	Sham acupuncture	Not reported	1. Sleep: PSQI decreased 2. Cognition: MoCA_B increased	China	1. Limited sample 2. No operator blinding 3. Lack long-term follow-up	Not reported
5.2	(63) Electroacupuncture	Not specified	36 cases	Subacute (3–12 months)	Electro group: 2 Hz continuous wave electroacupuncture Sham group: Simulated acupuncture at non-meridian points	Sham acupuncture	Not reported	1. Sleep: PSQI improved 2. Cognition: MoCA-B increased	China	1. Small sample 2. Short course (3 weeks) 3. No long-term follow-up	Not reported

This table summarizes acupuncture intervention modalities (body acupuncture, scalp acupuncture, electroacupuncture, auricular acupuncture, integrated acupuncture with herbal medicine) for PS-ICI. Columns specify acupoints/areas, intervention duration, primary outcome metrics (e.g., MoCA, PSQI, PSG sleep efficiency), and salient findings from included studies.

### 3.1 Body acupuncture therapy

Body acupuncture for PS-ICI has evolved from empirical point selection to syndrome-based targeted protocols, and its synergistic value has been increasingly documented in recent clinical investigations (11). The technique employs sterile stainless-steel filiform needles to stimulate meridian points; lifting-thrusting and rotating maneuvers are used to regulate qi and blood, exerting multi-target effects on PS-ICI (12). An RCT (13) enrolled 108 PS-ICI patients who were randomly assigned to an observation group (*n* = 54) or a control group (*n* = 54). The control group received routine Western medication, whereas the observation group was treated with Tongdu Jieyu (Governor-vessel depression-relieving) acupuncture plus Tongqiao Jieyu (Orifice-opening depression-relieving) decoction; treatment duration was not specified. Post-intervention, the observation group exhibited significant increases in Repeatable Battery for the Assessment of Neuropsychological Status (RBANS) cognitive scores and serum levels of dopamine (DA), norepinephrine (NE), and 5-hydroxytryptamine (5-HT), along with a significant reduction in PSQI scores (all *p* < 0.05); all indices improved to a significantly greater extent than in the control group (all *p* < 0.05). The observation group also demonstrated significantly higher effective-treatment rates and quality-of-life scores (all *p* < 0.01). These findings indicate that the combination of Tongdu Jieyu acupuncture and Tongqiao Jieyu decoction can simultaneously improve cognitive function and sleep quality in PS-ICI patients by modulating serum neurotransmitter levels, with efficacy superior to conventional Western therapy. Another study (14) included 60 PS-ICI patients who were randomly allocated to an experimental group (*n* = 30) or a control group (*n* = 30). Both groups received conventional stroke care; the control group was given routine acupuncture, while the experimental group received additional acupuncture at the five-zang back-shu points plus Bei-she points. Results showed that the experimental group had a significantly higher overall effective-treatment rate (*p* < 0.05), significantly higher MoCA scores, and significantly lower PSQI and AIS scores than the control group (all *p* < 0.05). In addition, the experimental group exhibited shorter sleep-onset latency, longer sleep maintenance time, and fewer nocturnal awakenings (all *p* < 0.05). These data suggest that acupuncture at the five-zang back-shu points combined with Bei-she points can significantly enhance cognitive function and sleep quality in PS-ICI patients, underscoring its clinical value.

### 3.2 Scalp acupuncture therapy

Scalp acupuncture integrates traditional Chinese meridian theory with contemporary cortical projection mapping to achieve targeted regulation of cerebral networks through precise stimulation of corresponding scalp regions (15). A multicenter randomized controlled trial (16) enrolled 445 PS-ICI patients who were randomly allocated to an experimental group (*n* = 223) or a control group (*n* = 222); both groups received conventional pharmacotherapy and rehabilitation exercise. The experimental group added interactive scalp acupuncture, whereas the control group received routine scalp acupuncture plus cognitive training. The intervention lasted 8 weeks, with an additional 2-month follow-up. At the end of the treatment period, the experimental group exhibited a 4.6-point increase in MoCA scores and a 5.2-point decrease in PSQI scores, while serum BDNF rose from 21.3 ± 5.6 ng·L^−1^ to 50.0 ± 7.2 ng·L^−1^; all improvements were significantly superior to those of the control group (all *p* < 0.01). These cognitive and sleep advantages persisted at the 2-month follow-up (all *p* < 0.01), indicating that interactive scalp acupuncture can simultaneously enhance cognitive function and sleep quality in PS-ICI patients within 8 weeks by up-regulating BDNF expression, with effects lasting at least 2 months post-intervention and outperforming routine scalp acupuncture combined with cognitive training. Zhang et al. (17) conducted another multicenter RCT that included 660 PS-ICI patients who were randomly assigned to Interactive Dynamic Scalp Acupuncture (IDSA), Simple Combined Therapy (SCT), or Traditional Scalp Acupuncture (TSA) groups, all receiving conventional medication and exercise rehabilitation for 8 weeks followed by a 2-month follow-up. Both post-intervention and follow-up assessments revealed that the IDSA group had significantly higher MoCA and Mini-Mental State Examination (MMSE) scores and significantly lower PSQI scores than the SCT and TSA groups (all *p* < 0.01), with greater magnitudes of improvement. This study confirms that IDSA, through the synergistic effect of scalp acupuncture and computerized cognitive training, can concurrently optimize cognitive function and sleep quality in comorbid patients, with stable efficacy over time, thereby offering a novel, precise clinical protocol for PS-ICI management.

### 3.3 Electroacupuncture therapy

Recent advances in electroacupuncture for PS-ICI indicate that low-frequency (2 Hz) electrical stimulation of Sishencong (EX-HN1) can simultaneously ameliorate sleep and cognitive deficits by modulating inter-regional brain connectivity: inhibiting activity in the right medial superior frontal gyrus facilitates sleep improvement, whereas enhancing activity in the left angular gyrus promotes cognitive recovery (18). Electroacupuncture involves inserting a filiform needle into the acupoint and delivering electrical current via the needle handle to intensify stimulation, thereby potentiating therapeutic efficacy—particularly for chronic pain and neurological disorders. Jiao et al. (19) conducted an RCT that recruited 36 PS-ICI patients who received either electroacupuncture at Sishencong or sham electroacupuncture for 4 consecutive weeks. After intervention, sleep efficiency increased by 22.3%, total sleep time was prolonged by 1.2 h, deep-sleep (N3) proportion rose by 15.4%, and REM sleep was extended by 8.7%. Cognitively, MoCA scores increased by a net 3.8 points, short-term memory accuracy improved by 25.1%, and reaction time was shortened by 18.2%. EEG revealed a 35.6% reduction in theta power (*p* < 0.001), indicating marked relief of hyper-arousal. Collectively, these multi-dimensional data demonstrate that electroacupuncture at Sishencong can optimize sleep architecture and enhance cognitive performance within 4 weeks in PS-ICI patients. A separate RCT (20) enrolled 60 PS-ICI patients who underwent 21 consecutive days of electroacupuncture at Sishencong (EX-HN1), while the pharmacological control group received estazolam tablets. Cognitively, the electroacupuncture group exhibited a net MoCA gain of 5.3 points (*p* < 0.01), significantly outperforming the estazolam group, which showed no significant change (*p* > 0.05); improvements in delayed recall, attention, and calculation sub-domains were 1.6 and 1.1 points, respectively (both *p* < 0.01). Sleep-wise, cardiopulmonary-coupling (CPC) analysis documented a 17% increase in sleep efficiency (*p* < 0.01), significantly superior to the 9% gain in the estazolam group (*p* < 0.05). Correlation analysis revealed a strong positive relationship between enhanced sleep efficiency and MoCA improvement (*r* = 0.88, *p* < 0.01), with significant associations also observed for delayed recall and language function. These findings confirm that electroacupuncture at Sishencong (EX-HN1) significantly improves both cognitive function and sleep efficiency in PS-ICI patients, with the two domains of improvement being closely inter-related.

### 3.4 Auricular acupuncture therapy

Auricular acupuncture regards the auricle as a microsystem of the human body; by stimulating specific auricular points it modulates the neuro-endocrine-immune network, producing analgesic, sedative, and anti-inflammatory effects that are simple to perform and minimally invasive (21). A published trial (22) combined auricular-point intervention (targeting Shenmen, cortex, and other core acupoints) with the traditional formula Qi-Yuan Decoction in 60 PS-ICI participants treated for 4 weeks, using estazolam tablets as a reference (30 subjects per group). Cognitively, the intervention group showed pronounced improvement in MoCA scores (*p* < 0.05), with significant gains in attention, delayed recall, and calculation domains. Sleep-wise, total PSQI scores decreased markedly, sleep efficiency increased, and sleep-onset latency shortened; some data indicated efficacy non-inferior or even superior to estazolam (*p* < 0.05), yielding overall benefits comparable to or greater than conventional medication. Mechanistic studies revealed a more pronounced reduction in serum cortisol levels in the intervention group (*p* < 0.05), attributed to down-regulation of hypothalamus–pituitary–adrenal (HPA)-axis activity and consequent attenuation of hippocampal damage, resulting in synergistic improvement of both sleep and cognition. Current evidence indicates that auricular acupuncture combined with traditional Chinese herbal formulas can provide dual sleep–cognitive benefits for individuals with PS-ICI.

### 3.5 Integrated acupuncture and herbal medicine therapy

Acupuncture–herbal combination therapy represents a comprehensive strategy that simultaneously or sequentially applies the two core techniques of traditional Chinese medicine—acupuncture and herbal prescription—to achieve synergistic enhancement, reduce drug dosage, and minimize adverse effects. In an RCT conducted by Sui et al. (23), 80 PS-ICI patients were randomly assigned to a combination group or a control group (40 cases each); both groups received conventional Western medicine, while the combination group was additionally treated with Tiaoren-Tongdu (governor-vessel–regulating) acupuncture plus Huoxue-Jieyu (blood-stasis–dispelling depression–relieving) decoction for 12 weeks. After treatment, LOTCA scores increased in both groups, whereas PSQI, State–Trait Anxiety Inventory (STAI) scores, and serum dopamine (DA) levels decreased, and serum 5-HT levels increased (all *p* < 0.05). Moreover, the magnitude of improvement in LOTCA, PSQI, and STAI, as well as the rise in 5-HT and the reduction in DA, were significantly greater in the combination group than in the control group (all *p* < 0.05). The study confirms that acupuncture combined with herbal medicine can markedly promote cognitive recovery, ameliorate sleep quality and anxiety, and effectively modulate serum 5-HT and DA neurotransmitter levels in PS-ICI patients.

Existing studies confirm that multiple acupuncture modalities can improve cognitive and sleep functions in patients with PS-ICI; however, the magnitude of MoCA score improvement varies slightly across protocols, which is related to acupoint specificity and treatment duration. Key limitations of current research remain prominent: unclear source tracing in some reports, lack of standardized operating procedures and systematic adverse-event monitoring; IDSA is highly dependent on professional skills and specialized equipment, and its synergistic mechanism with cognitive training has not been clarified, making wide promotion difficult. Herbal formulas such as Qi-Yuan Decoction and Huoxue-Jieyu Decoction, as well as acupuncture–herb combined regimens, are markedly affected by batch variability and regional restrictions; for example, Qi-Yuan and Huoxue-Jieyu decoctions are only available in China. Long-term longitudinal studies and factorial designs are needed to disentangle the independent effects of acupuncture and herbal medicine.

## 4 Neurobiological regulation of neural signaling pathways

**Figure 2:**
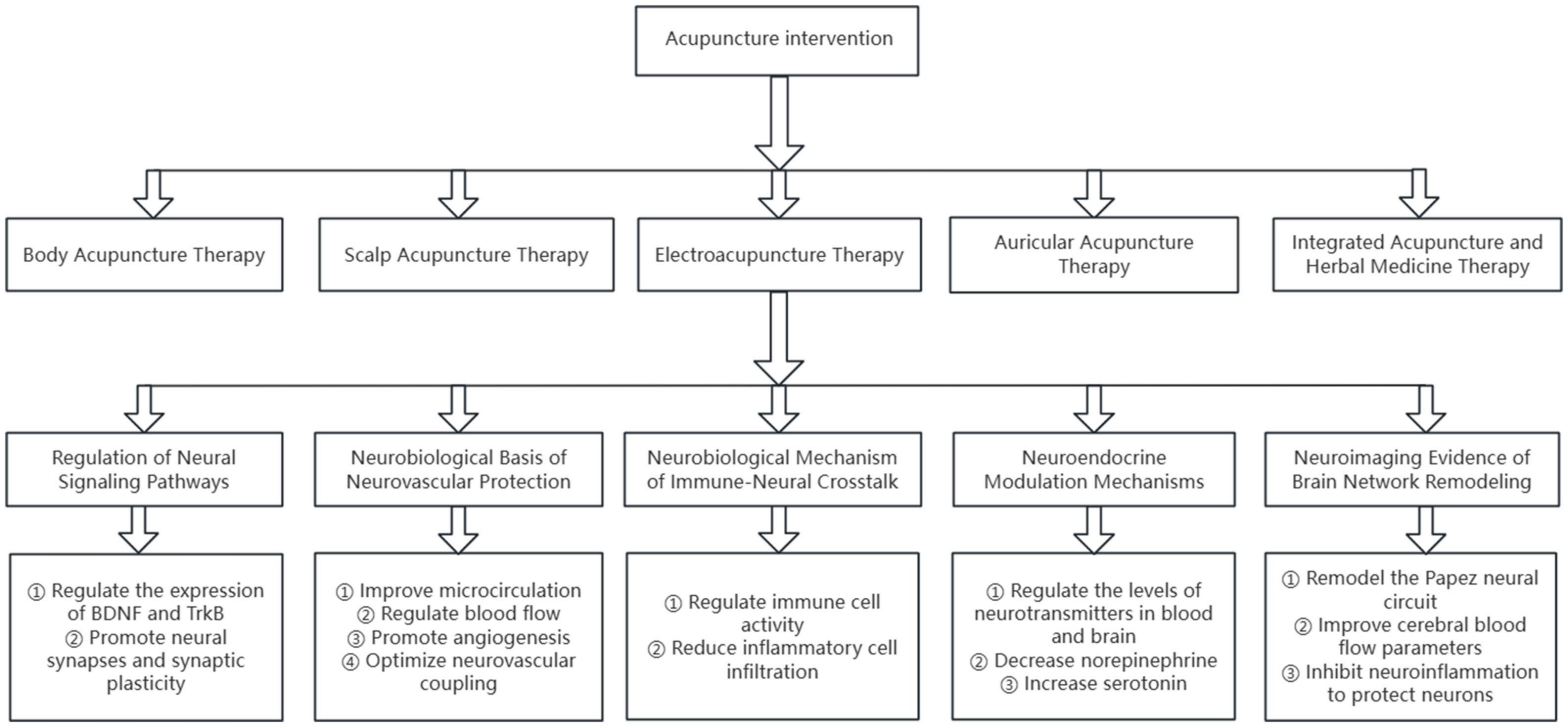
Schematic of Acupuncture’s Mechanisms for PS-ICI Treatment. This figure illustrates the mechanisms of acupuncture for treating PS-ICI: (1) Regulation of Neural Signaling Pathways, (2) Neurobiological Basis of Neurovascular Protection, (3) Neurobiological Mechanism of Immune-Neural Crosstalk, (4) Neuroendocrine Modulation Mechanisms, (5) Neuroimaging Evidence of Brain Network Remodeling. Together, they promote cognitive and sleep function recovery in PS-ICI patients.

The mechanisms of action of acupuncture in treating PS-ICI are complex and diverse, involving multiple levels such as the regulation of neural signaling pathways, neurovascular protection, immune modulation, and brain network regulation. These mechanisms interact with each other and act synergistically to collectively promote the recovery of patients’ cognitive and sleep functions (Figure 2).

### 4.1 Regulation of neural signaling pathways

Recent studies have expanded the neuro-signaling modulation of acupuncture for PS-ICI to the mitogen-activated protein kinase (MAPK) pathway, including its core subtypes c-Jun N-terminal kinase (JNK) and extracellular signal-regulated kinases (ERK). These modules synergize with the canonical BDNF/PI3K/Akt cascade to amplify the coordinated sleep–cognition improvement by suppressing neuroinflammation and reducing neuronal apoptosis (24). Acupuncture up-regulates BDNF and its receptor TrkB, thereby activating the PI3K/Akt signaling axis (25). As a pivotal regulator of neuronal growth and synaptic plasticity, BDNF elevation after cerebral ischemia inhibits neural apoptosis, promotes cell survival, and facilitates the regeneration and remodeling of injured neurons via this pathway (26), while also being intimately linked to central nervous system disorders such as ischemic stroke and Alzheimer’s disease (27). Electroacupuncture increases hippocampal BDNF and TrkB protein expression and PI3K/Akt phosphorylation in cerebral ischemia/reperfusion rats, reducing infarct volume by 34.6%, improving neurological function and behavioral indices, and enhancing spatial learning and memory (28). Yin et al. (29) randomized 90 PS-ICI patients into acupuncture plus repetitive transcranial magnetic stimulation (rTMS), rTMS alone, or medication (one dropout each) for 4 weeks. The medication group received oral escitalopram; the rTMS group added 20 Hz rTMS; the combined group added governor-vessel-regulating and spirit-harmonizing acupuncture (points: Baihui, Sishencong, etc.). Post-intervention, the acupuncture plus rTMS group exhibited lower HAMD-17 and PSQI scores and higher MoCA scores and serum 5-HT and BDNF levels than the other groups (all *p* < 0.05 or *p* < 0.01), indicating that elevating serum 5-HT and BDNF simultaneously improves cognition and sleep in PS-ICI. Liu et al. (30) randomized 80 PS-ICI patients into an acupuncture group (*n* = 40, estazolam plus harmonizing acupuncture) or an acupuncture-plus-herb group (*n* = 40, plus self-formulated depression-relieving sour jujube decoction) for 2 weeks. The combined group showed prolonged total sleep time, reduced PSQI scores and sleep latency, and higher MoCA scores (all *p* < 0.05), along with elevated serum NGF, BDNF, and 5-HT, and decreased norepinephrine (NE) and neuropeptide Y (NPY) levels (all *p* < 0.05). The regimen activates the NGF-BDNF pathway to repair neurons and balances monoaminergic neurotransmitters, synergistically improving cognition and sleep, and providing evidence-based support for acupuncture intervention in PS-ICI at the neural-signal-pathway level.

Although acupuncture has been shown to modulate the BDNF–TrkB-PI3K/Akt pathway in humans (31), existing studies are predominantly small-scale, single-center trials that lack standardized, multicenter validation, resulting in limited evidence quality. Moreover, mechanistic investigations have largely focused on isolated pathways, leaving the cooperative regulatory nodes between the MAPK and BDNF–TrkB-PI3K/Akt cascades undefined. Intervention protocols and acupoint-stimulation parameters remain heterogeneous, constraining the translational value of these findings.

### 4.2 Neurobiological basis of neurovascular protection

Recent advances demonstrate that acupuncture not only down-regulates neuronal injury biomarkers such as neuron-specific enolase (NSE) and S100 calcium-binding protein B (S100β) but also concurrently repairs the sleep–cognitive regulatory network by improving cerebral microcirculation and modulating cranial-nerve-branch function, offering a non-pharmacologic intervention for PS-ICI that is supported by both efficacy and mechanism (32). Acupuncture enhances microcirculation, regulates cerebral blood flow, promotes angiogenesis, and optimizes neurovascular coupling to synergistically increase brain-tissue perfusion (33). Its protective effects further include modulation of astrocytic function to preserve blood–brain barrier (BBB) integrity, creating a stable microenvironment for neural repair (34). Zhang et al. (35) enrolled 88 PS-ICI patients (sleep comorbidity: sleep-apnea syndrome) and randomized them into two groups receiving routine Western therapy for 3 weeks. The observation group (*n* = 44) received additional acupuncture at Lianquan (CV23) once daily before sleep, whereas the control group (*n* = 44) received Western therapy alone. Results showed that the observation group exhibited an 8.05% increase in sleep efficiency and a 50.9% prolongation of sleep latency, with superior sleep-index improvements (all *p* < 0.05); serum NSE and S100β levels decreased by 33.7 and 13.7%, respectively, and MoCA scores increased by 1.82 points (26.67 ± 2.90 vs. 24.85 ± 3.02), yielding a total clinical effective rate of 93.18% versus 77.27% in controls (all *p* < 0.05). Mechanistically, acupuncture at CV23 stimulates the glossopharyngeal and hypoglossal nerve branches, improves pharyngeal muscle tone and airway patency, down-regulates neuronal injury biomarkers, and significantly enhances sleep and cognitive function in PS-ICI patients. Shi et al. (36) randomized 122 PS-ICI patients into two groups for a 30-day intervention. The trial group (*n* = 62) received transcranial ultrasound combined with scalp acupuncture (Baihui GV20, Sishencong EX-HN1, horizontal insertion 1 cun, 30-min retention), whereas the control group (*n* = 60) received transcranial ultrasound alone. The trial group achieved a PSQI total score of 7.6 ± 1.0 (vs. 12.1 ± 3.5 in controls) and a reduction in NIHSS scores from 28.91 ± 0.29 to 19.80 ± 0.37, with both indices superior to controls (all *p* < 0.05). Mechanistically, scalp-needle stimulation regulates governor-vessel qi, sedates, and tranquilizes, synergistically improving cerebral microcirculation with transcranial ultrasound to concurrently relieve sleep and cognitive disturbances in PS-ICI patients.

Although current clinical studies have demonstrated the efficacy of acupuncture for PS-ICI, several methodological limitations remain prominent: most trials have not incorporated sham or placebo controls, lack blinded designs, and exhibit insufficient methodological rigor; sample sizes are small, intervention periods are short, and long-term follow-up data are absent, making it difficult to verify sustained therapeutic or neurovascular protective effects (37); additionally, heterogeneity in intervention protocols across studies has prevented the establishment of unified standards, thereby limiting the generalizability of the findings.

### 4.3 Neurobiological mechanism of immune-neural crosstalk

Recent studies have clearly shown that acupuncture exerts multi-target immune regulation in PS-ICI by centering on the gut–brain-axis remodeling (38), neurogenic inflammation suppression (39), and circadian immune rhythm recovery (40)—an immune–neural crosstalk that provides new theoretical support for the simultaneous improvement of cognition and sleep. Acupuncture modulates immune-cell activity to reduce inflammatory infiltration and attenuate the inflammatory response (41); it regulates cytokine expression to suppress inflammatory signaling pathway activation; and it tunes immune-organ function to enhance systemic immunity (42). These synergistic mechanisms mitigate inflammatory damage and correct immune imbalance. In a prospective study, Bian et al. (43) enrolled 128 elderly PS-ICI patients and randomly assigned them to two groups (*n* = 64 each): the control group received estazolam, while the observation group received estazolam plus Tiaoshen-Xingnao (mind-regulating and brain-refreshing) acupuncture for 4 weeks. Outcomes including efficacy, sleep, neurological function, cognition, and serological indices were compared. The observation group achieved a higher total effective rate, exhibited reduced PSQI, AIS, and NIHSS scores, elevated MoCA scores, shortened sleep-onset latency, increased sleep efficiency and N3 proportion, prolonged total sleep time and REM sleep duration, and higher serum 5-HT and NPY levels with lower substance P (SP) levels (all *p* < 0.05). Mechanistically, this regimen synchronously improves sleep and cognition in elderly PS-ICI patients by modulating serum neurotransmitters. Zhang et al. (44) prospectively enrolled 85 PS-ICI patients and randomly allocated them: 42 to the control group receiving butylphthalide and 43 to the study group receiving butylphthalide plus Tiaoshen-Yiqi (mind-regulating and qi-tonifying) acupuncture. Sleep, functional, and serological indices were compared. The study group demonstrated significantly longer total sleep time, higher sleep efficiency, better Fugl-Meyer Assessment (FMA) and MoCA scores, and elevated serum 5-HT and GABA levels, along with significantly fewer awakenings, shorter sleep-onset latency, lower PSQI and neurological deficit scale scores, and reduced serum hs-CRP and TNF-*α*levels versus the control group (all *p* < 0.05). Mechanistically, this protocol up-regulates neurotransmitters to enhance sleep regulation while suppressing inflammation factor-mediated neuronal injury, concurrently optimizing sleep and cognitive function in PS-ICI patients.

Accumulating evidence indicates that acupuncture down-regulates systemic inflammatory markers (hs-CRP, TNF-α) while up-regulating central neurotransmitters (5-HT, GABA) in patients with PS-ICI through immune–neural interactive modulation, with gut–brain-axis remodeling emerging as a pivotal mechanistic direction. Nevertheless, the specific molecular links that mediate synergistic sleep–cognitive improvement and the corresponding cross-pathway regulatory nodes remain undefined. Current data are largely limited to static correlations and lack dynamic longitudinal tracking (45).

### 4.4 Neuroendocrine modulation mechanisms

Up-to-date neuroimaging evidence confirms that acupuncture enhances functional connectivity and synaptic plasticity within the Papez circuit (46), thereby strengthening synchronous regulation of the HPA-axis–monoaminergic rhythm axis and providing direct circuit-level support for acupuncture-mediated brain-network remodeling and concurrent sleep–cognitive improvement in PS-ICI patients (47). Acupuncture modulates serum neurotransmitter levels by lowering norepinephrine and elevating 5-HT, which improves sleep architecture, alleviates insomnia symptoms (48), enhances sleep quality, and promotes cognitive recovery (49). Li et al. (50) recruited 216 PS-ICI patients for an RCT; compared with healthy controls, patients exhibited a 32% reduction in MoCA scores (*p* < 0.001) and a 38% increase in PSQI scores (*p* < 0.001), along with 1.8-fold and 2.1-fold up-regulation of interleukin-6 and tumor necrosis factor-*α*, respectively, indicating that concurrent sleep–cognitive dysfunction is closely related to neuroinflammation. After 12 weeks of manual acupuncture at ST36, GV20, and SP10 (twice weekly, 30 min per session), patients showed a 29% improvement in MoCA scores (*p* = 0.002), a 31% decrease in PSQI scores, and inflammatory factor levels that returned to approximately 85% of healthy-control values; the magnitude of cognitive improvement was significantly positively correlated with sleep-quality gains and the degree of inflammation suppression (all *p* < 0.01). These results demonstrate that acupuncture can markedly ameliorate cognitive impairment and sleep disturbance in PS-ICI patients by modulating acetylcholine–5-HT neurotransmitter balance and suppressing neuroinflammation. Zhou et al. (51) randomized 220 PS-ICI patients into two groups: the control group received routine Western medical therapy, while the trial group was treated with Huang-Lian Wen-Dan decoction combined with acupuncture for 12 weeks. The results showed that the trial group achieved a significantly higher total effective rate than the control group (*p* < 0.05). In addition, serum levels of BDNF, 5-HT, andβ-amyloid 1–42 (Aβ1-42), as well as MoCA scores, were significantly elevated, whereas AIS scores and homocysteine (HCY) levels were significantly reduced (all *p* < 0.05). The study confirmed that the combined intervention not only repairs neuronal damage and improves cognitive function via 5-HT, but also synergistically enhances sleep-regulatory pathway signaling by increasing 5-HT levels and reduces the neurotoxic effects of HCY, thereby markedly improving both cognitive and sleep function in PS-ICI patients.

Neuroimaging evidence has demonstrated that acupuncture can enhance Papez circuit function and modulate the HPA-axis–monoaminergic rhythm axis; however, the causal relationship between simultaneous improvements in 5-HT and cortisol rhythms and increased brain-network connectivity remains unclear. Furthermore, differences in acupuncture efficacy among patients with different Traditional Chinese Medicine syndromes and stroke stages have yet to be defined (52).

### 4.5 Neuroimaging evidence of brain network remodeling

Recent neuroimaging and resting-state electroencephalography studies have verified that acupuncture can modulate functional connectivity within brain networks of PS-ICI patients, including the dynamic balance of the DMN, salience network (SN), and central executive network (CEN), as well as frequency-specific connections in relevant brain regions (53). These findings provide direct visual evidence for clarifying the neural circuit mechanisms underlying simultaneous sleep–cognitive improvement (54). Compared with patients suffering from ischemic stroke alone, individuals with PS-ICI exhibit triple imbalance across DMN, SN, and CEN: functional connectivity of the posterior cingulate–hippocampus pathway is significantly reduced, and DMN integrity disruption impairs memory consolidation (55); abnormally enhanced insula–thalamus coupling induces persistent hyper-arousal of SN (56), leading to insomnia; node efficiency in the dorsolateral prefrontal cortex decreases by 22%, causing insufficient executive resources in CEN and resulting in working memory deficits. Acupuncture concurrently reduces abnormal DMN–SN coupling and significantly enhances CEN node efficiency, reshaping the “resting–salience–executive” dynamic balance and ultimately achieving synergistic improvement of cognition and sleep (57). Through multi-target modulation of the Papez circuit, acupuncture improves cognitive function by remodeling the hippocampus–mammillary body–anterior thalamic nucleus–cingulate pathway and simultaneously ameliorating cerebral hemodynamic parameters (58). In a randomized controlled trial (59), 196 PS-ICI patients were equally allocated to an observation group and a control group (*n* = 98 each). The control group received conventional care plus rehabilitation training combined with hyperbaric oxygen (HBO) therapy, whereas the observation group was additionally treated with “Zhi-san-zhen” (wisdom-three-needle) acupuncture for 28 days. Compared with controls, the observation group exhibited significantly higher MoCA and LOTCA cognitive scores and greater P300 wave amplitudes, along with a more pronounced increase in serum BDNF and IGF-1 levels; concurrently, PSQI and AIS sleep scores were significantly lower, and serum VILIP-1, homocysteine (Hcy), and oxidative-stress indices were significantly reduced (all *p* < 0.05). No difference in complication rates was observed between groups (*p* > 0.05), confirming that HBO plus “Zhi-san-zhen” acupuncture can achieve dual cognitive–sleep benefits in PS-ICI patients by modulating neurotrophic factors and ameliorating oxidative stress, with an acceptable safety profile. Although studies of acupuncture-mediated brain-network modulation and combined interventions have yielded initial insights, the specific neural-circuit targets of “Zhi-san-zhen” remain undefined, and cutting-edge techniques have yet to be employed to dissect the micro-mechanisms underlying DMN functional remodeling (60).

Existing studies have confirmed that acupuncture modulates PS-ICI through multi-dimensional mechanisms—including neural signal pathway regulation, neurovascular protection, immune–neural crosstalk, neuroendocrine modulation, and brain network remodeling—yet several core limitations persist: the causal cross-talk among these multi-system regulatory pathways remains unclear, and an integrated theoretical framework for “multi-mechanism synergy” is lacking; clinical studies generally lack long-term neurobiological dynamic tracking data, making it difficult to verify the sustainability of regulatory effects; moreover, hierarchical validation of acupuncture parameter effects is insufficient, and individual heterogeneity effects on intervention outcomes have not been adequately addressed. Therefore, multi-time-point, multi-modal mechanistic validation and clinical translation studies are urgently needed.

## 5 Imaging studies on acupuncture treatment for PS-ICI

### 5.1 Structural magnetic resonance imaging studies

Structural magnetic resonance imaging (sMRI) captures morphological changes in brain tissue through high-resolution T1-weighted imaging, providing objective evidence for assessing neural structural damage in PS-ICI. An RCT (61) enrolled 72 PS-ICI patients and randomly assigned them to an electroacupuncture (EA) group, a sham electroacupuncture (SA) group, and a healthy control (HC) group. Both groups received routine rehabilitation, while the EA group additionally received 2 Hz low-frequency electroacupuncture at Sishencong (EX-HN1), and the SA group received non-acupoint sham needling. The results showed that PSQI, MoCA, SAS, and SDS scores in the EA group were significantly better than those in the SA group after treatment, confirming that the protocol can simultaneously improve sleep, cognition, and negative emotions. fMRI analysis showed that electroacupuncture could specifically regulate key brain regions: it inhibited the overactivation of brain regions such as the right medial superior frontal gyrus (SFGmed. R) and the right precuneus (PCUN), and enhanced the functional activity of the left angular gyrus (ANG. L) and the left inferior parietal lobule (IPL. L); among them, the functional connectivity (FC) of SFGmed. R was positively correlated with PSQI scores, suggesting that constraining its activity is one of the core mechanisms for improving sleep. In addition, electroacupuncture can reduce the lateral connection efficiency between the whole brain and the thalamus and hippocampus, making the function of abnormal brain regions approach the physiological state, showing a reconstruction feature of “dominant hemisphere enhancement and non-dominant hemisphere weakening.” These evidence confirms that low-frequency electroacupuncture at Sishencong can simultaneously improve sleep, cognitive and emotional states in PS-ICI patients by regulating key nodes and functional connections of brain networks.

### 5.2 Voxel-based morphometry

Voxel-based morphometry (VBM) is a core quantitative technique of structural magnetic resonance imaging that can precisely capture microstructural changes in gray-matter density of brain regions, providing objective evidence for the neurobiological mechanisms of acupuncture intervention (62). Wang et al. (63) conducted a randomized controlled trial of 36 PS-ICI patients and found that the gray-matter density of sleep-cognition-related brain regions was significantly higher in comorbid patients than in healthy controls, with lower MoCA scores and higher PSQI scores (*p* < 0.05), suggesting that gray-matter structural abnormalities are a key pathological basis for PS-ICI. After 3 weeks of electroacupuncture intervention at Sishencong with 2 Hz continuous wave (20 min per session), VBM revealed a specific reduction in gray-matter density in the left lingual gyrus and other visual signal processing brain regions, accompanied by increased MoCA scores and decreased PSQI scores (*p* < 0.05), while no significant changes were observed in the sham acupuncture group. This study indicates that electroacupuncture can reduce cortical visual signal processing sensitivity by modulating gray-matter microstructure remodeling in cognition- and sleep-related brain regions, thereby simultaneously improving post-stroke sleep and cognitive function, providing direct high-grade structural imaging evidence for acupuncture treatment of PS-ICI (64).

Using multimodal sMRI and VBM imaging, this chapter constructs a full “macro-structural repair → micro-structural remodeling” evidentiary chain that systematically clarifies the neuro-radiological mechanisms underlying acupuncture for PS-ICI. Critical limitations remain, however: the cross-level linkage between molecular indices (BDNF, neurotransmitters) and imaging parameters is undefined, long-term longitudinal data are scarce, and stratified validations of different acupuncture parameters on core imaging metrics and molecular targets are missing, all of which constrain mechanistic depth and clinical translatability.

## 6 Discussion

Acupuncture has garnered converging support from both clinical and pre-clinical investigations for the management of PS-ICI. At the clinical level, 4 weeks of electro-acupuncture at Sishencong (EX-HN1) increases deep-sleep (N3) proportion by 15.4% and elevates MoCA scores by 3.8 points. Mechanistically, these effects are underpinned by modulation of synaptic plasticity via the BDNF–TrkB–PI3K/Akt cascade, suppression of neuro-inflammation through the brain–gut axis, and re-organization of DMN connectivity (65, 66)—together forming a “traditional-to-modern” tri-dimensional evidence chain that uniquely restores the reciprocal sleep–cognitive network (67). The core TCM concept of “regulating Shen and re-animating the brain” aligns intrinsically with these contemporary findings: “regulating Shen” corresponds to re-balancing DMN hyper-arousal and aberrant functional connectivity, thereby stabilizing sleep rhythms and executive performance, whereas “re-animating the brain” reflects BDNF–TrkB–PI3K/Akt-mediated neuro-plastic repair that promotes hippocampal synaptogenesis and circuit re-instatement, ultimately reversing cognitive decline and sleep-architecture disruption. This acupuncture-mediated modulation thus offers a modern interpretation of classical doctrine and furnishes a translational bridge between Eastern and Western therapeutics (68). Of note, preliminary data suggest that distinct TCM syndrome sub-types (e.g., Heart–Spleen deficiency, Liver–Kidney Yin deficiency) map onto divergent neuro-biological phenotypes; however, evidence supporting syndrome-differentiated acupuncture remains insufficient (69), underscoring a critical direction for future inquiry.

Current research is constrained by four core limitations that must be dissected at the fundamental level. First, mechanistic studies remain fragmented, with a lack of cross-system synergistic logic, thereby limiting translational value. The molecular regulatory mechanisms underlying the coordinated interaction between “synaptic plasticity–immune rhythm–brain–gut axis” remain unclear; for instance, direct evidence is lacking regarding the temporal correlation between BDNF upregulation and NF-κB-mediated anti-inflammatory effects, as well as the regulatory mechanisms by which gut microbiota-derived metabolites modulate the BBB tight-junction protein ZO-1 (70, 71). Most animal models adopt a single “middle cerebral artery occlusion (MCAO) + sleep deprivation” paradigm (72), which differs from the complex pathological processes observed in clinical PS-ICI patients, namely “ischemia–sleep disturbance–cognitive decline–emotional dysfunction” (73), making it difficult to directly translate mechanistic conclusions (74). Second, clinical studies lack standardization and subtype adaptability. Existing studies are limited by small sample sizes (mostly <300 cases), short follow-up durations (mostly ≤24 weeks), and the absence of unified standards for electroacupuncture frequencies (2/100 Hz vs. 50 Hz) and acupoint combinations (75); moreover, most studies have inadequate blinding reporting, potentially introducing bias, and individualized dose-adjustment thresholds for short-term cognitive suppression induced by high-frequency electroacupuncture remain undefined (76). Qi-Yuan decoction, Shu-Gan-Jie-Yu decoction, and other herbal formulations are restricted to East-Asian populations, lack dosing algorithms for the chronic phase, and require cross-regional validation (77); the association between TCM syndrome subtypes and neurobiological phenotypes has not been tested in large cohorts, leaving the modern biological footing of syndrome-based acupuncture unsecured (78). Third, multi-dimensional evidence integration is insufficient and sham-acupuncture controls are methodologically flawed. Current studies rely on single-omics readouts (serum cytokines, fMRI) (79), omitting integrated analyses of m^6^A epigenetic regulation or protein–protein interactions such as PSD-95–synaptophysin coupling (80, 81), thereby obscuring the full spectrum of acupuncture’s multi-scale modulatory effects. Moreover, sham protocols usually employ “non-meridian superficial needling” that may still activate neuroactive pathways, introducing placebo noise and impeding precise discrimination between specific and non-specific effects of acupuncture (82). Fourth, long-term efficacy and safety data are scarce, with key endpoints unquantified: 1-year cognitive retention, stroke recurrence risk, and disease-modifying impact on neurodegeneration remain unmeasured, limiting confidence in the long-range utility of acupuncture for post-stroke rehabilitation (83).

Future investigations must achieve targeted breakthroughs against the outlined limitations by focusing on four interlocked priorities, thereby forging a “limitation→breakthrough” closed-loop logic that moves the field from “evidence accumulation” to “precision evidence-based practice.” Priority one is to decode cross-system synergies and dismantle mechanistic fragmentation. Leverage single-cell RNA sequencing and spatial transcriptomics to define the molecular grammar governing the “synaptic plasticity–immune rhythm–brain–gut axis” crosstalk (e.g., the temporal coupling between BDNF up-regulation and NF-κB-mediated anti-inflammatory signaling) (84); employ human brain-organoid and organ-on-a-chip models that integrate ischemia, sleep disturbance, and mood dysfunction to interrogate Notch pathway dynamics; and apply integrated microbiome–metabolome profiling to clarify how gut-microbial metabolites regulate the tight-junction protein ZO-1 at the blood–brain barrier (85), thereby constructing a multi-system-integrated mechanistic network. Second, advance clinical standardization and subtype-specific research. Launch multicenter, large-scale RCTs (≥500 participants) to establish a three-dimensional “stroke-stage–TCM-syndrome–intervention” matching standard (86): recommend “Xing-Nao-Kai-Qiao” acupuncture plus cognitive training in the acute phase and adopt a “core body-acupuncture formula plus syndrome-based modification” in the chronic phase. On the basis of current efficacy and safety data (87), 20 Hz can serve as a reference frequency for PS-ICI electroacupuncture; future head-to-head RCTs should validate its superiority over other frequencies (e.g., 2 Hz, 100 Hz) in predefined responder subgroups, while individualized dose-adjustment thresholds are derived from baseline MoCA scores with fMRI-guided precise acupoint selection (88). Third, intensify multi-technology fusion and multi-omics integration to refine sham-acupuncture controls. Perform joint epigenomic, proteomic and fMRI analyses to uncover m^6^A methylation patterns and PSD-95–synaptophysin interactions (89). To overcome inherent sham-needling flaws, implement a triple-control design (non-meridian location + no de-qi sensation + depth <2 mm) and verify—via functional neuroimaging—the absence of specific neural activation in the sham group, thereby minimizing placebo confounds (90). Fourth, initiate long-term follow-up and cross-regional validation to fill the evidentiary gap. Design studies with ≥1-year follow-up to quantify 1-year cognitive retention, stroke recurrence risk and disease-modifying effects on neurodegeneration (91); Extend recruitment to multi-ethnic and geographically diverse cohorts to validate the cross-population generalizability of the herbal formulation, and to delineate acupuncture parameter adaptations optimized for Western populations (92).
